# Knowledge structures of scientific production on COVID-19 in the sphere of education: the case of publications indexed in the Web of Science during 2020

**DOI:** 10.1007/s11135-022-01564-w

**Published:** 2022-10-28

**Authors:** Clemente Rodríguez-Sabiote, Álvaro Manuel Úbeda-Sánchez, Oswaldo Lorenzo-Quiles, José Álvarez-Rodríguez

**Affiliations:** 1 Department of Methodology in Research and Diagnostic in Education, University of Granada, Granada, Spain; 2 Department of Methodology in Research and Diagnostic in Education, University of Granada, Melilla, Spain; 3 Department of Department of Didactics of Musical, Plastic and Body Expression, University of Granada, Melilla, Spain; 4 Department of Pedagogy, University of Granada, Granada, Spain

**Keywords:** Knowledge structures, COVID-19, Educational sphere, Bibliometric analysis, Scientific mapping

## Abstract

This study seeks to explore the different knowledge structures in the sphere of educational research into COVID-19 during 2020. Using bibliometric methods, analysis was performed of a sample of 308 scientific articles retrieved from the Web of Science database. Using different data analysis techniques combining co-occurrence analysis, co-citation analysis and factorial analysis, All Keywords and Keywords Plus were used to achieve the main research objective: identification of the main themes and trends of production in the sphere of educational research into COVID-19. The main findings of this study in terms of the conceptual structure show that analysis of the centrality and density of the thematic trends points to a generalised structural change in the entire educational system towards methodological teaching–learning procedures oriented towards distance education. As for the intellectual structure, among the host of authors and sources of information involved only a select few have a greater influence on the scientific community. Finally, in terms of social structure, there is limited collaboration between authors and institutions from different countries. However, this collaboration is more intense within countries themselves and in terms of their own production, with the USA being the country with the strongest links.

## Introduction

Since the COVID-19 disease caused by the SARS-CoV-2 virus became a worldwide pandemic during 2020, it has posed a major challenge for a number of business and production sectors, not to mention many individuals and their families in particular. In this uneasy climate, a variety of problems have arisen that include loss of employment, lack of income, social uncertainty, mobility restrictions and, of course, the severe effect on public health due to the high infection rate of the virus and the collapse of many public health systems which are a priori duly prepared for all kinds of contingencies (Chahrour et al. 2020). Since the outbreak the economies of many countries have been seriously affected, which is why considerable financial and human resources have been mobilised in record time to find a solution in the form of a vaccine (Burton and Walker 2020; Caddy 2020) that is capable of alleviating the situation and at the same time generating greater confidence among the population (Harrison and Wu 2020). In many countries, various strategic vaccination programmes have already begun. Despite this, the health situation continues to be high-risk, mainly due to two aspects: the slow execution of the vaccination process in the majority of territories, partly due to failure by pharmaceutical companies to comply with the terms initially agreed for manufacture and distribution of the vaccine and also because in many territories the first vaccines have not even arrived.

**Fig. 1:**
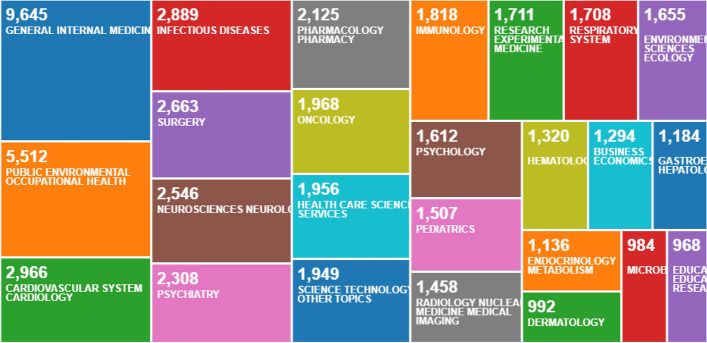
Tree Map of top 25 COVID-19 research areas in 2020 with search by ‘title’ on Web of Science

**Fig. 2:**
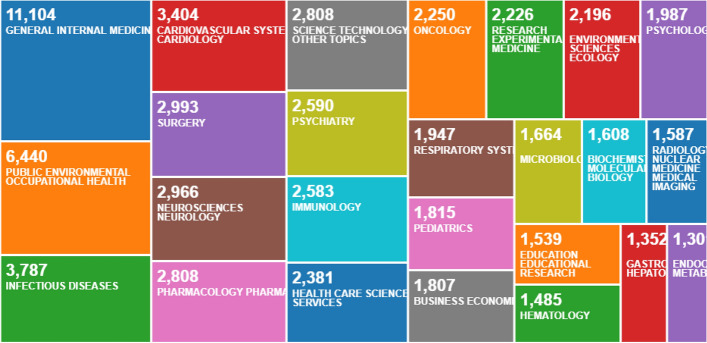
Tree Map of top 25 COVID-19 research areas in 2020 with search by ‘topic’ on Web of Science

In this context, most of the different scientific disciplines have focused a large proportion of their research studies on COVID-19 throughout 2020. Logically, medical research in general is the discipline that covers this topic of worldwide interest to the greatest extent detriment to increasingly common educational research. (Corell et al. 2021; Waltz Comarú et al. 2021). However, a series of searches in the Core Collection of the Web of Science database shows that education is among the top 25 areas of research into COVID-19. More specifically, Fig. 1 shows that based on a search using the term ‘covid-19’ by ‘title’ for the year 2020, the area of Education and Educational Research is ranked 25th with 968 records out of a total of 61,032. This positions it as the second most important area for research into COVID-19 in the field of Social Sciences (excluding all medical science disciplines), solely behind Business Economics. Similarly, Fig. 2 shows the same search parameters using the ‘topic’ search type, with Education and Educational Research ranking 22nd with 1539 records out of 76,540, again second only to Business Economics in terms of areas unrelated to medical research.

This highlights the significant capacity of educational research to address the major structural changes that it may undergo. Throughout this period of uncertainty, the education system and teachers and students in particular have undergone a rapid shift towards new modes of teaching–learning processes from early childhood right through to university level (Allen et al. 2020), mainly centred on distance or online education. This last aspect is undoubtedly one of the main focal points of changes in educational practice and also an area where research has placed special emphasis. There is abundant literature on the new digital era in the educational world, with some authors already considering online learning as a panacea in times of crisis due to COVID-19 (Dhawan 2020). However, it must also be taken into account that this new, technology-intensive educational situation may leave many students in a vulnerable situation and thus give rise to digital inequalities that were previously concealed from view (Beaunoyer et al. 2020; Drane et al. 2021; Jaeger and Blaabaek 2020).

Faced with these changes and the new realities that have abruptly altered educational practice and research interests, we set out with a general interest in the different knowledge structures relating to COVID-19 in the field of educational research. These were determined by means of science mapping, a strategy the fundamental task of which is to find representations of intellectual connections within the dynamically changing system of scientific knowledge (Small 1997). In other words, science mapping aims to display the structural and dynamic aspects of research in the different scientific areas (Aria et al. 2022; Börner et al. 2005; Morris and Van Der Martens 2008; Franceschini and Maisano 2012; Khaldi and Prado-Gascó 2021). For Aria and Cuccurullo (2017) and Lorenzo et al. (2022) Science Mapping allows investigating scientific knowlegde from a statistical point of view configured around: Conceptual structure: What science talks about, the main; Intellectural Structure: How the work of an author influences a given scientific community and Social Structure: how authors, institutions and countries interact each other conceptual structure represents relations among concepts or words in a set of publications.

Conceptual structure refers to the relationships between concepts or words in a set of publications (Aria and Cuccurullo 2017). In this context, words that appear together in a document will be related in a co-words network (López-Belmonte et al. 2021). The conceptual structure is therefore useful for understanding the topics covered by a field of research and defining which are the most important and the most recent (research front). It can also be useful for studying the evolution of topics over time. In addition to network analysis, a fundamental strategy in the conceptual structure is factor analysis, which is very useful for identifying thematic dimensions. The different typologies of factor analysis will be described in more detail in Sect. 4.2 below. Finally, we find the mixed approach within the so-called conceptual structure. Starting from a conceptual network, thematic networks are identified by means of a graph representing the centrality and density of the thematic network. The concepts of centrality and density will be discussed in more detail in Sect. 4.3.

Moreover, we also find the intellectual structure. The main purpose of this structure is to show the different relationships between the nodes that represent the bibliographic references under analysis (Nguyen et al.^2021^). In this structure we highlight that the network edges can have different interpretations depending on the citation type (co-citation or direct citation). Two types of analysis shape the intellectual structure. One hand, there are the co-citation networks (Small 1973) and, on the other hand, the historiographic mapping (Garfield 2004).

Finally, we will look at social structure. Through the social structure we can see how authors or institutions relate to others in the field of scientific research. The social structure is materialised through the co-authorship network (Peters and Van Raan 1991). The usefulness of this network is to offer information about which are the most influential authors, the most relevant institutions in the different fields of research, hidden communities of authors, etc.

## Research objectives

We have based our research objectives on the various scientific knowledge structures as follows:

1. To define the main themes that may be considered trends in scientific production on COVID-19 in the field of education during 2020 (conceptual structure).
2. To determine how the different articles published by various authors on the topic of research into COVID-19 influence the educational scientific community (intellectual structure).
3. To determine how the different authors who have published articles on COVID-19 in the field of education interact with the various institutions and countries to which they belong and how these institutions and countries interact among themselves (social structure).

## Methods

### Study variables

The three variables studied are related to three different dimensions: conceptual structure, intellectual structure and social structure.

With regard to the first dimension, conceptual structure, this is based on the relationships between concepts or words belonging to a group of scientific publications. Words included together in a scientific paper can form different networks, i.e. co-word networks, based on the relationships between these words. These structures can be useful to understand the different themes that make up the various research fields and thus determine what the hot topics or research fronts are.

In relation to the second dimension, intellectual structure, work has been carried out on the basis of co-citation networks, taking two documents cited jointly as the basis and establishing a link between nodes. According to the edges of the networks, different interpretations can be made depending on the type of citation between authors or documents (co-citation or direct citation). This structure can be very useful to detect shifts in paradigms of thinking.

Finally, the third dimension, social structure, is characterised by analysis of social relations to establish scientific collaboration networks in a given field. It is based on joint authorship of publications, incorporating centrality indicators to allow identification of the main actors in the network, with classification into three levels: authors, institutions and countries.

### Sampling

The Core Collection of the Web of Science (WoS) was used to compile the data that make up the sample. The reason for choosing WOS over SCOPUS is that, although the latter database includes a larger number of journals, the citation analysis in WoS is more detailed (Falagas et al. 2008). In conclusion, we can say that WoS covers a larger time span, with the majority being English-language journals. SCOPUS covers a larger number of lower impact journals (Kulkarni et al. 2009). The articles were retrieved from a search of the Web of Science database on 1 February 2021. The search was filtered by ‘title’ using the search terms ‘educat*’ and ‘covid-19’ together with the Boolean operator ‘and’. The search was restricted to the year 2020 and refined according to the four thematic categories contemplated by the database for education: Education and Educational Research; Education in Scientific Disciplines; Special Education and Educational Psychology. Finally, papers of an empirical nature were selected, in this case articles already published and early access articles, resulting in a final sample of 308 scientific articles.

## Results

For the analysis of the information selected via the Web of Science (WoS) database, we have relied on the RStudio v.4.0.4 programmes using the Bibliometrix package in a more user-friendly environment, namely the Biblioshiny interface (Aria and Cuccurullo 2017); and VOSviewer v.1.6.16 (Van Eck and Waltman 2020).

### Conceptual structure

Firstly, for the configuration of the conceptual structure we have mapped the co-occurrence network of papers on COVID-19 related with education and published in scientific journals indexed in the Web of Science during the course of 2020. For this first analysis we have considered both Author Keywords and Keywords Plus, i.e. all keywords, with the idea of including in this conceptual structure the main themes in both their more particular and general scope without any type of refinement.

The use and justification of keywords plus as an analysis element for the factorial approach and thematic maps is based on the fact that they are terms generated by the Web of Science database itself from the titles of the articles cited, being terms that appear more than once in the bibliography and ordered based on phrases ranging from several words to simple terms. This is a more traditional retrieval of titles and keywords in a more standardised and generic way than author keywords, with a more specific and particular emphasis which would not provide us with the more global and general vision that is truly sought in this particular analysis. In addition, the Biblioshiny interface also poses a limitation in that it allows content analysis to be carried out using author keywords, keywords plus and also terms inferred from the titles of the articles as well as their abstracts, but always in isolation, which makes it impossible to carry out combinations of joint analyses.

### Co-occurrence network

In order to determine the visual structure of the co-occurrence network that can be derived from the documents under analysis, we have used the following calculation parameters via the VOSviewer → Parameters programme: Type of analysis: Co-occurrence; Unit of analysis: All keywords; Counting method: Full counting; Network Layout: Automatic Layout; Clustering Algorithm: Association strength; Minimum number of occurrences of a keyword: 5 (of the 789 keywords, 63 meet the thresholds). In addition, Graph Theory was used to interpret the results, which is essentially a mathematical discipline that focuses on the study of graphs, mathematical structures used to model the pairwise relationships between objects. A graph is composed of vertices (also called nodes or points) that are connected by edges (also called links or lines). The Network visualisation based on all keywords of the documents shown below is based on a multi-loop undirected multigraph where the edges link two vertices symmetrically.

As can be seen in the network visualisation based on all keywords of documents, various groups or clusters have been inferred with greater internal cohesion that distance them from the rest of the clusters, and which are identified using different colours. The size of the label and the node of an item are further determined by the weight of that item. In this particular case, it can be seen how the most important all keywords were *covid-19*; *education*; *internet/web-based learning*; *curriculum*; *second-year undergraduate*; *first-year undergraduate/general*; *distance learning/self instruction* and *laboratory instruction*. On the other hand, the distance between the different all keywords in the network visualisation roughly indicates their relationship in terms of co-occurrence links. For each of the 63 keywords, the total strength of co-occurrence links with other keywords is calculated. In this way, the keywords with the highest overall link strength are selected. The following table shows the 20 most relevant keywords based on their co-occurrence values and total link strength (Table 1).

It can be seen that one of the main keywords, covid-19, with an occurrence value of 77, is not one of the terms with the highest link strength, with a value of 101. This may be due to the fact that in the cluster to which it belongs (red cluster) the rest of the terms or nearest neighbouring nodes are more focused on medical aspects and the virus itself, such as *medical students*; *coronavirus*; *medical education* or *sars*. Although related to educational research in this case, they are not the main research area that can be inferred. However, we did find a direct relationship between the keywords relating to distance learning and the changes experienced in the curriculum (the first five keywords in Table 2) as the nodes with the highest occurrence values and also the strongest links. This shows how the major structural change that education has been obliged to urgently address, and in which educational research has shown its main interest, is the adaptation of the curriculum and teaching–learning procedures to new educational scenarios with a high presence of new technologies and the internet.

**Table 1:** Main sample information

Description	Results
Documents	308
Sources (only journals)	28
Keywords Plus (ID)	314
Author Keywords (DE)	512
Period	2020
Collaboration Index	4.39
Article	278
Article: early access	30

**Table 2:** Occurrence between the 20 most relevant all keywords and total link strength

Keyword	Occurrences	Total link strength
Distance learning/self instruction	92	471
First-year undergraduate/general	64	318
Internet/web-based learning	59	304
Second-year undergraduate	50	286
Curriculum	44	234
Upper-division undergraduate	40	218
Laboratory instruction	36	193
Student-centred learning	33	179
Organic chemistry	28	160
Education	35	153
Computer-based learning	27	151
General public	27	128
Professional development	24	109
Collaborative/cooperative learning	20	108
Multimedia-based learning	19	105
Interdisciplinary/multidisciplinary	20	103
Covid-19	77	101
Inquiry-based/Discovery learning	18	96
Biochemistry	15	75
Chemistry	13	75

In addition to the network visualisation, the item density visualisation is shown in the following figure.

There are two variants of density visualisation; the first is item density visualisation and the second is cluster density visualisation. As observed by Van Eck and Waltman (2010), it should be taken into account that in the item density visualisation, items are represented by their label in a similar way to the network visualisation and the overlay visualisation. Each point in the item density visualisation has a colour indicating the density of items at that point. By default, the colours vary from blue to green and finally to yellow. The greater the number of items in the vicinity of a point and the greater the weight of the neighbouring items, the closer the colour of the point is to the yellow-shaded areas. Conversely, the lower the number of items in the vicinity of a shady zone or point and the lower the weight of the neighbouring items, the closer these items are to the blue-shaded areas. We therefore find that the cluster to which the term *covid-19* belongs is a dense item located in the middle of the area shaded in yellow, although the rest of the items of the cluster are located in more peripheral areas corresponding to the green and blue colours with a lower density. On the other hand, terms such as *internet/web-based learning*; *second-year undergraduate*; *first-year undergraduate/general* or *distance learning/self instruction* occupy the yellow zones of their respective clusters, which have a higher general density. Furthermore, although in the network map it can be seen how the *curriculum* item belongs to the blue cluster, it is a very relevant item on its own as it has a high density and considerably high values for occurrence and total link strength despite not having other neighbouring nodes in its vicinity.

Having carried out the most visual and descriptive mapping possible, we continued with the characterisation of the conceptual structure of scientific production on COVID-19 in the educational sphere and indexed in the Web of Science during 2020, using the factorial approach as an analytical resource. From this point onwards, the analyses have been carried out based on the keywords plus.

Essentially, the factorial approach allows a large set of variables to be reduced to latent components or factors, in other words, reduction of the dimensionality of the data for their representation in a space with lower dimensionality. Aria and Cuccurullo (2017) and Cuccurullo et al. (2016) considered three variations to the factorial approach, namely: Correspondence Analysis (CA), Multiple Correspondence Analysis (MCA) and Multidimensional Scaling (MDS). With Husson et al. (2017) we can say that Correspondence Analysis (CA) is an extension of principal component analysis suited to explore relationships among two qualitative variables (or categorical data). Like principal component analysis, it provides a solution for summarizing and visualizing data set in two-dimension plots. Multiple correspondence analysis (MCA), on the other hand, can also be understood as a generalization of *Correspondence Analysis* (CA) to the case where there are more than two variables (Husson and Josse 2014). Finally, Multidimensional Scaling (MDS) is a visual representation of distances or dissimilarities between sets of objects. The general aim of multidimensional scaling is to find a configuration of points in a space, usually Euclidean, where each point represents one of the objects or individuals, and the distances between pairs of points in the configuration match as well as possible the original dissimilarities between the pairs of objects or individuals (Cox and Cox 2008).

This study has used the MCA factorial approach, as our work has focused on exploring a large number of qualitative variables based on keywords plus of documents obtaining the following results in relation to the conceptual structure map.

For the interpretation of the conceptual structure map, it must be taken into account that the proximity between words corresponds to a shared communality. Thus, if keywords plus appear close to each other in the conceptual structure map (areas or clusters of terms in blue and red) it is because a large proportion of articles include them together. Conversely, these terms are distant from each other when only a small number of articles use these words together.

In this manner, the conceptual structure map shows the formation of two large groups of well-differentiated keywords plus in which the explained variances in both dimensions amount to 41.58% for dimension 1 (*x*-axis), which provides the greatest explanation, and 19.54% for dimension 2 (*y*-axis), which is less relevant. On the one hand there is a blue cluster (on the left-hand side of the map) and another red cluster (on the right-hand side of the map).

The first cluster (blue) is made up of a total of 18 keywords plus, most of which are related to what could be called the *chemistry science* component. In fact, it should be taken into account as an initial premise that positions close to the origins of the *x*-axis *(x* = *0*) and *y*-axis *(y* = *0*) represent the median position of all column profiles and therefore the centre of the research field, i.e. the most important commonly shared themes. Thus, we can see that this blue cluster is positioned on coordinates closer to both origins with keywords plus such as *chemistry*, *general chemistry*, *design*, *science*, *knowledge*, etc. in dimension 1 and terms such as *food, curriculum, skills, education* and *video* in dimension 2. Precisely, all these keywords plus may be considered as relevant topics commonly dealt with in the scientific papers analysed.

On the other hand, the red cluster on the right-hand side of the map has 7 keywords plus located very far from the origin of dimension 1 and, on the contrary, with more keywords plus close to the origin of dimension 2. This cluster could be referred to as the educational component based on the methodologies and evaluation of the teaching–learning process. It can be seen that this cluster is less important than the blue cluster, since it is only explained by dimension 2.

In short, it may be affirmed that dimension 1 of the conceptual structure map differentiates between two well-defined components: keywords plus of greater weight *vs.* lesser weight in the scientific literature relating to COVID-19 in the education sphere. Meanwhile, dimension 2 of the conceptual structure map seems to discern between the keywords plus related with the *chemistry science* component and the educational component.

### Thematic map

To conclude the characterisation of the conceptual structure, the following is the thematic map corresponding to the following initial parameters: The field used is Keywords Plus using a maximum of 250 words and a minimum cluster frequency of 3 words. For each cluster a single tag corresponding to the most frequent keyword is used.

**Fig. 3:**
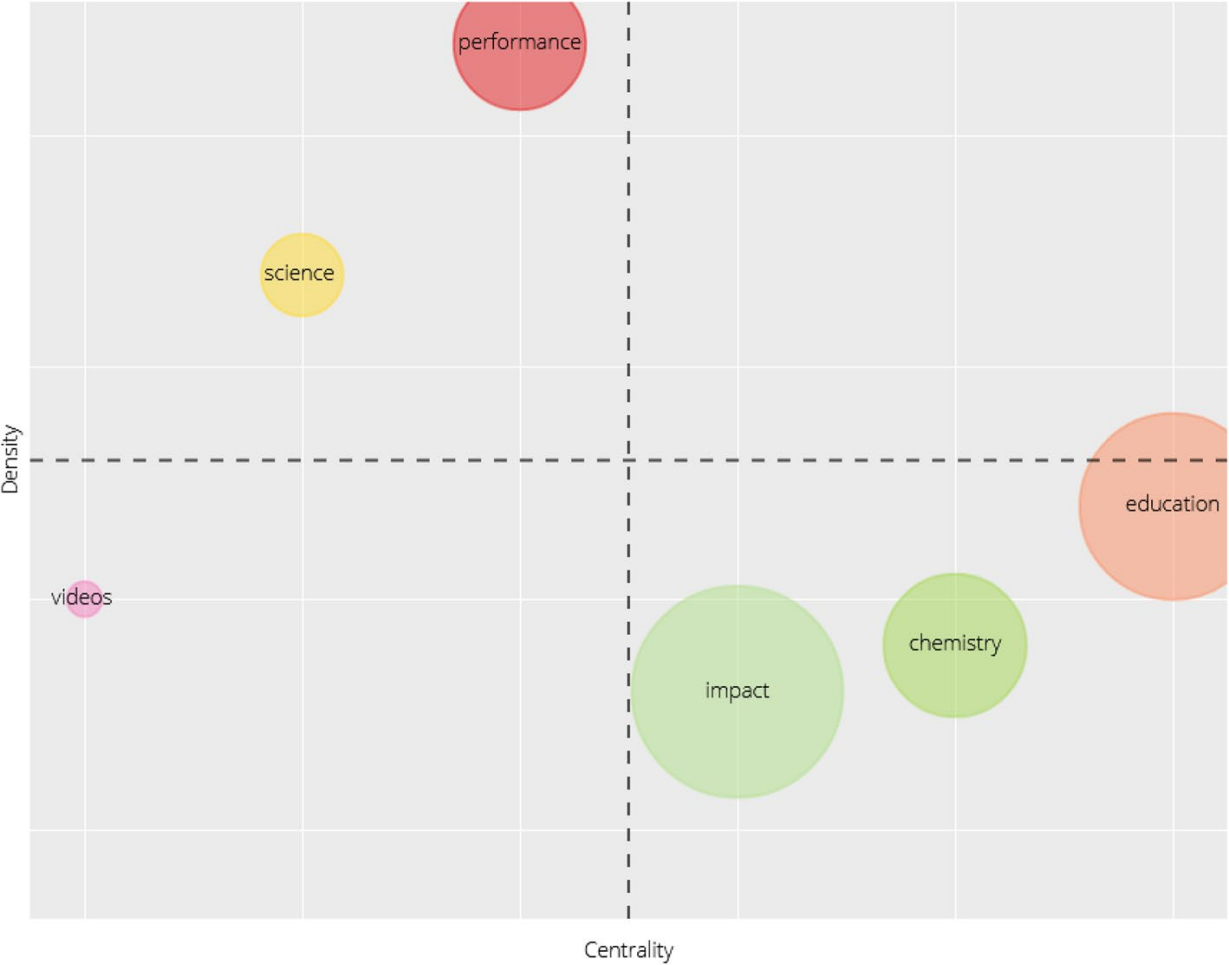
Thematic Map based on keywords plus of documents

In the thematic map set out in Fig. 3, each bubble represents a network cluster, while the bubble labels represent the keyword with the highest occurrence value. The size of each bubble is proportional to the number of keyword occurrences in the cluster and its positioning on the map is established according to centrality and density measurements. Centrality is understood as being a global measurement providing information on the position that a node occupies in relation to other nodes (De la Rosa Troyano et al. 2005). This measurement may be interpreted as the degree of importance of a theme within a particular research area (Giannakos et al. 2016). The density is a local measurement that indicates the position of a node with respect to a set of neighbouring nodes, and may be used to explain the level of development of a theme (Cobo et al. 2001). To interpret the thematic map we have followed the indications of Cobo et al. (2001), dividing the four quadrants of the Cartesian axis into upper left quadrant for *highly developed and isolated themes* (quadrant 3); lower left quadrant for *emerging or declining themes* (quadrant 4); upper right quadrant for *motor themes* (quadrant 1); and lower right quadrant for *basic and transversal themes* (quadrant 2).

Looking at Fig. 3, it can be seen that there is no bubble to indicate that any theme can be considered a *motor theme*. In this particular case, the *video*s bubble would be considered an *emerging theme* with low centrality, null density and an occurrence value of 6, which may be related due to its nature and context to the new types of distance education. The *science* and *performance* bubbles correspond to *highly developed and isolated themes* in the main area being considered of educational research and COVID-19, with a strong presence of technology and virtual environments. Both these bubbles have a high centrality that translates into a higher external connectivity with other nodes. In the case of *science*, it has a null density solely composed of the keyword *science* with an occurrence value of 5, while *performance* has a relative density, that is, an internal connectivity between its three keywords *performance*, *curriculum* and *online* with occurrence values of 11, 6 and 5 respectively. Finally, we have the *impact*, *chemistry* and *education* bubbles relating to *basic and transversal themes*. The three bubbles have little centrality with the exception of *education*, which has a relative centrality perhaps because it is the most generic and cross-cutting of the three themes. In terms of density *education* has the highest levels, not so much in terms of the degree of internal connectivity between its keywords but rather in terms of the occurrence value of its terms. Of its three keywords, *education* has an occurrence of 25, *students* with 12 and *video* with 5. The *chemistry* bubble has another three keywords, with *chemistry* having an occurrence value of 13, *skills* with 6 and *lab* with 5. The last bubble corresponds to *impact* and is the one with the greatest internal connectivity in terms of the number of keywords it contains. Up to eight keywords were identified with the following occurrence values: *impact* with 11, *classroom* with 9, *technology* with 8 and *engagement*, *flipped classroom*, *student performance*, *clickers* and *general-chemistry* with 6.

### Intellectual structure

The intellectual structure shows the relationships between nodes representing bibliographic references. In this scenario, the network edges can have different interpretations depending on the type of citation, namely whether it is co-citation or direct citation. In bibliometric tradition, it is more common to use the strategy of co-citation between authors, documents or any other chosen parameter (Small 1973; McCain 1991). Co-citation analysis is based on the premise that “between two or more documents that are co-cited (cited together) in a third and subsequent work, there is, at least from the citing author’s perspective, a thematic similarity; and the greater the frequency of co-citation, the greater the affinity between them” (Miguel et al. 2007, p. 142). For the arrangement of the intellectual structure we have based ourselves on the determination of the co-citation network, taking as a reference three initial differentiated parameters: authors, sources and organisations. The results obtained by the three co-citation networks inferred are presented below.

#### Intellectual structure based on authors

The network parameters used are based on the field of authors using the strength of association of their links. The network design is automatic and the minimum number of citations of an author is 10 (out of the 4430 authors, 26 meet the threshold).

#### Intellectual structure based on sources

The network parameters used are based on the field of sources using the strength of association of their links. The network design is automatic and the minimum number of citations of a source is 10 (of the 2456 authors, 55 meet the threshold).

#### Intellectual structure based on organisations

The network parameters used are based on the field of organisations. The network design is automatic and the clustering algorithm is Louvain. The maximum number of nodes is 50.

**Fig. 4:**
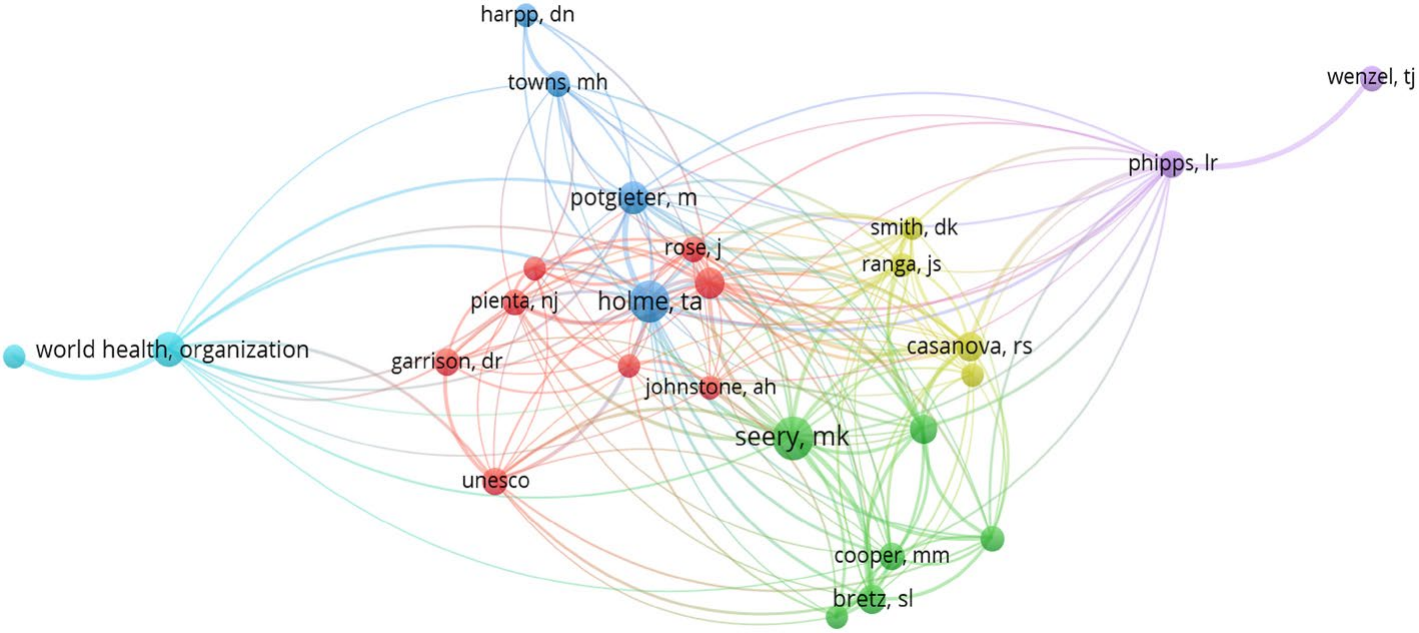
Co-citation network based on the field ‘authors’

**Fig. 5:**
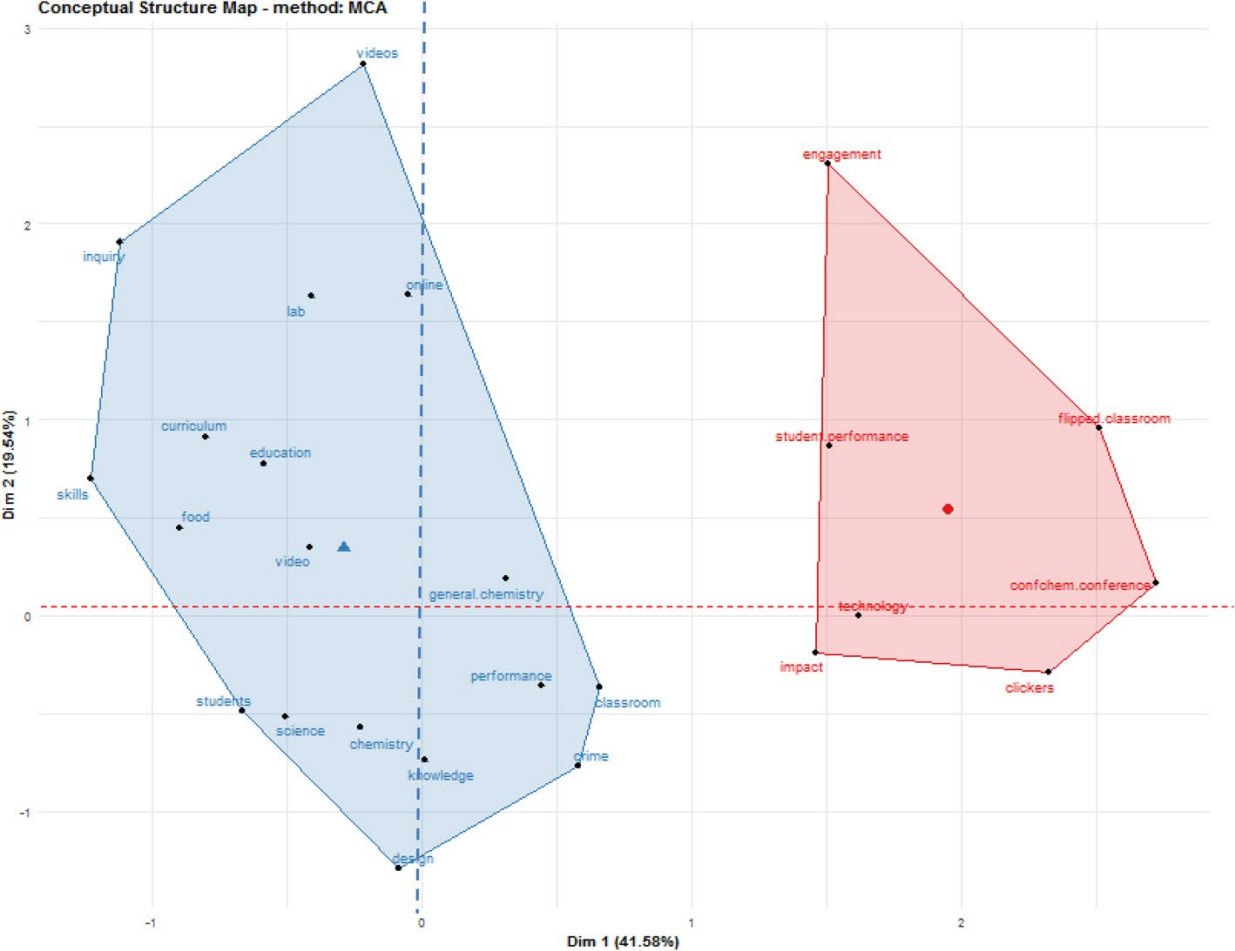
Conceptual structure map (MCA method) based on keywords plus of documents

**Fig. 6:**
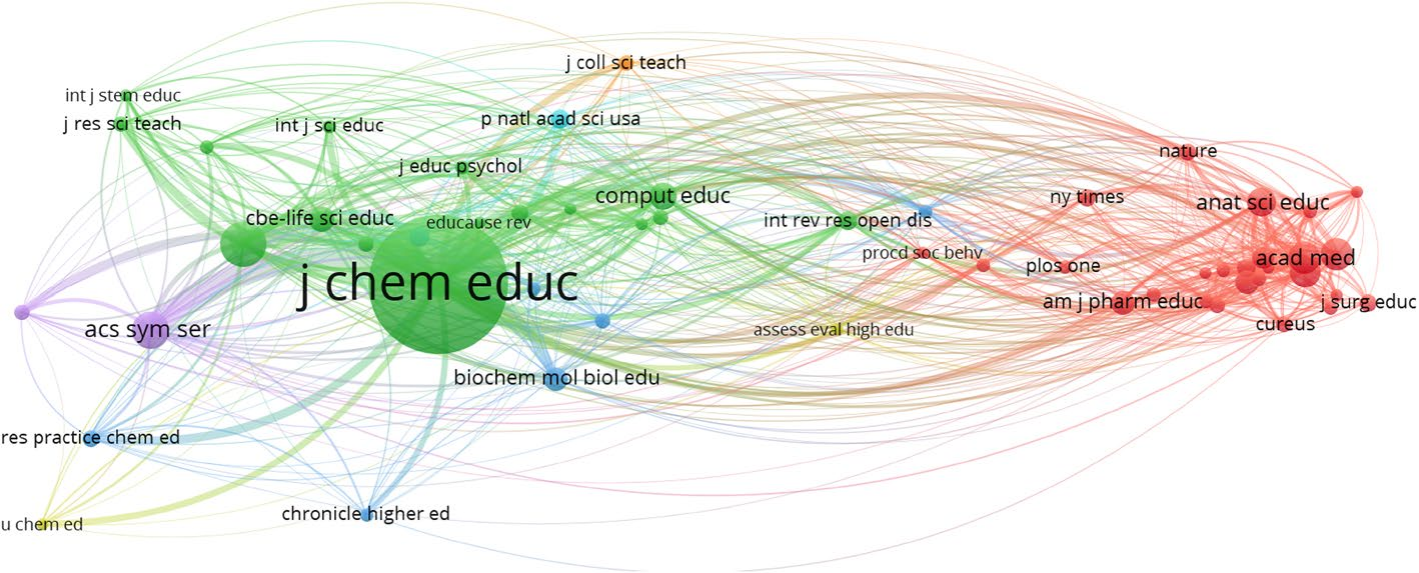
Co-citation network based on the field ‘source’

**Fig. 7:**
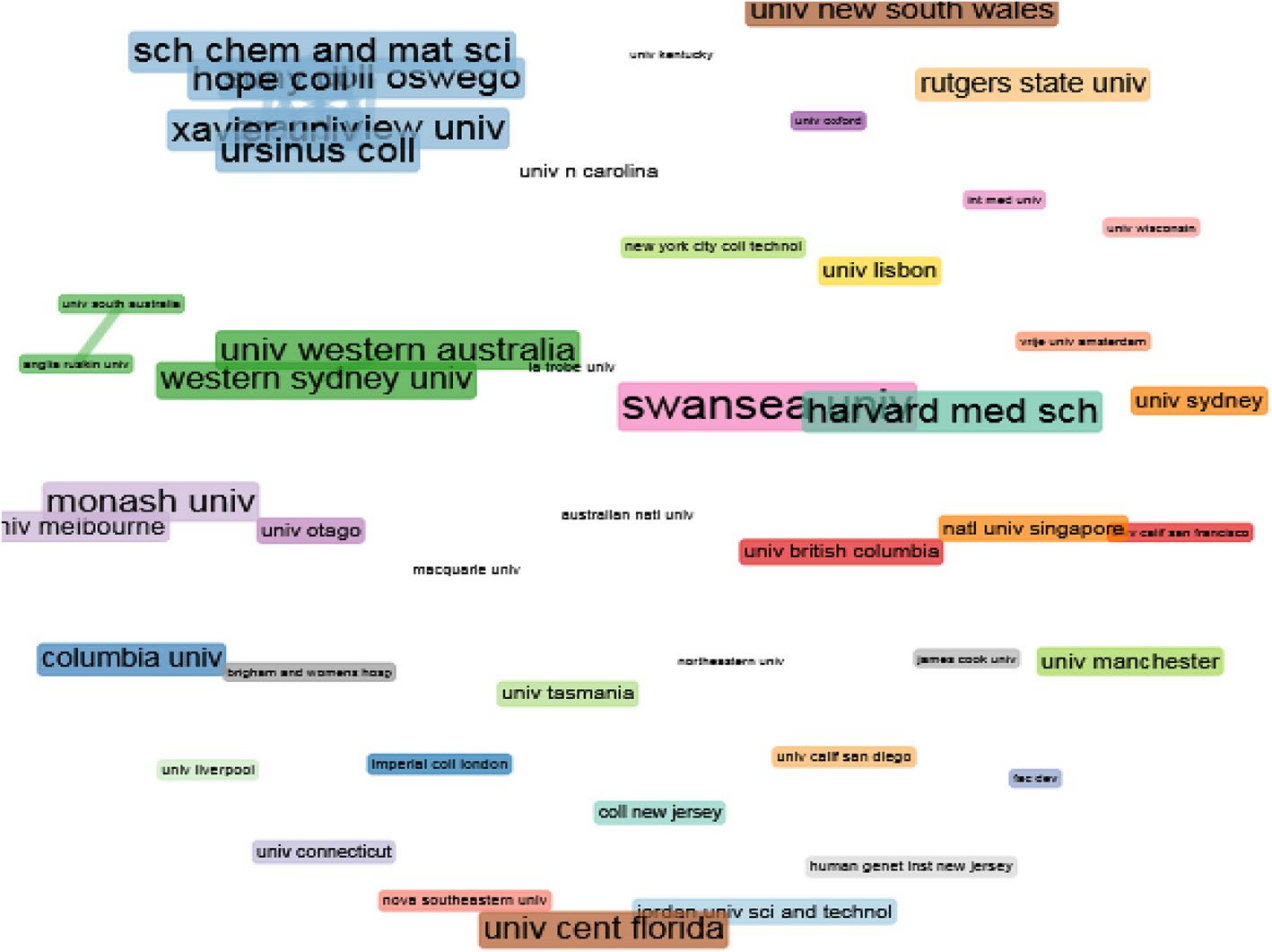
Co-citation network based on the field ‘organisation’

From a detailed evaluation of Figs. 4, 5, 6 and 7 that make up the intellectual structure, first of all we can see that there is a fairly rich network in terms of co-citation between authors. It is true that Fig. 4 shows only 26 authors with the highest number of co-citations, with a minimum of 10 co-citations per author for a total of 4430 authors. However, there are fairly strong relationships and proportionality between all authors. In the central part of Fig. 4, it is worth noting the formation of various associations characterised by different colours, among which we find certain authors that stand out from the others. By colours, M. Potgietery and T. A. Holme lead the blue cluster. These two authors, together with M. H. Towns and D. N. Harpp, have in common the fact that they stand out from the rest, having been cited during 2020 in works where they address the convenience of establishing new strategies for teaching–learning of chemistry that are adapted to exceptional situations such as those experienced during the current COVID-19 pandemic. M. K. Seery heads a second important cluster (green cluster) together with M. M. Cooper and S. L. Bretz. All three authors have in common that they have been cited in papers relating to procedures and tasks for the assessment of chemistry as a discipline during the COVID-19 pandemic. There is also a third cluster (red cluster) made up of various authors, including N. J. Pienta, D. R. Garrison and J. Rose, etc., who are cited in works that continue to focus on chemistry teaching and learning methodologies such as the flipped classroom. There are also other smaller more residual clusters further away from the centre, such as the one formed by the WHO (World Health Organization) on the left-hand side of the figure. All the cases appear to faithfully reflect the well-known principles of Lotka’s Law (1926), whereby most authors receive a lower number of co-citations while a few authors have the highest values of co-citations.

The same is true for the sources cited (Fig. 6), where the red cluster shows very important relationships and strong links due to the concentration of sources more closely related to medical and/or pharmacological research. However, it is led by the *Journal of Chemical Education,* with a node size far above the rest of the green cluster and which consequently has greater centrality and external connectivity with the rest of the neighbouring nodes (or sources), thus fulfilling the aforementioned theory propounded by Lotka (1926). In addition, we can see how the *Journal of Chemical Education* makes up an important satellite network of co-citations based on the field ‘source’ with various journals, whose common characteristic is study of the relationship between chemistry and education. This result was foreseeable, if we take into account that the main co-citation networks based on the ‘authors’ field are related to the teaching–learning process of chemistry in COVID-19 times.

As for the co-citation network based on organisations (Fig. 7), there are no outstanding relationships or co-citation networks based on the ‘organisation’ field and there is a clear and notable lack of cooperation between organisations. The dispersion between institutions is truly noteworthy, with only small nuclei of co-citation networks based on the ‘organisation’ field at intra-national level, for example, the case of the purple cluster between the University of Melbourne, Monash University and Otago University in the Australian-New Zealand context; the green cluster made up of University of Western Australia, Western Sydney University, etc., for the Australian case alone; and, finally, the case of Ursinus College, Hope College, Xavier University, etc. for the North American (USA) case.

### Social structure

The social structure shows how authors or institutions relate to each other in a given scientific field of research. In this context, the most commonly envisaged way of elaborating the social structure is based on co-authorship networks (Peters and Van Raan 1991). In essence, the authorship network refers to the joint authorship of a scientific paper by two or more authors. We have relied on different parameters to determine the various social structure pathways to be inferred, as in the previous cases with the conceptual and intellectual structures.

#### Social structure based on authors

The network parameters used are based on the field of authors using the strength of association of their links. The network design is automatic and the maximum number of authors per document is 25 and the minimum is 2 (of the 1081 authors, 34 meet the threshold).

#### Social structure based on organisations

The network parameters used are based on the field of organisations using the strength of association of their links. The network design is automatic and the maximum number of organisations per document is 25 and the minimum is 2 (of the 398 organisations, 79 meet the threshold).

#### Social structure based on countries

The network parameters used are based on the field of countries using the strength of association of their links. The network design is automatic and the maximum number of countries per document is 25 and the minimum is 5 (of the 57 countries, 10 meet the threshold).

**Fig. 8:**
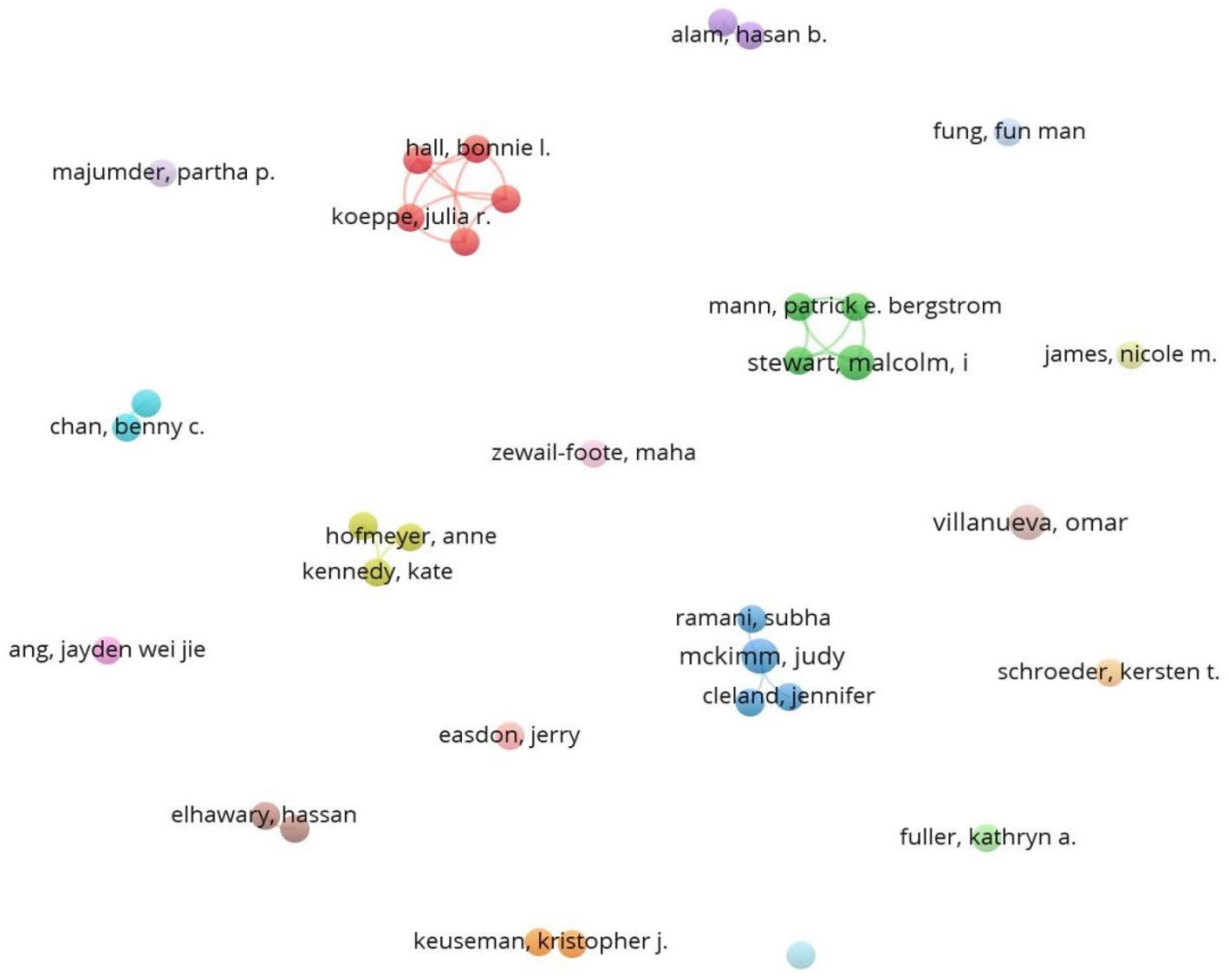
Co-authorship network based on the field ‘authors’

It can be seen that there is little collaboration between authors (Fig. 8). There are clusters that show collaborations between four or five authors, although none of them stand out above the rest with significant production as indicated by the size of the nodes in Fig. 8, although the minimum established in the parameters is two documents per author.

**Fig. 9:**
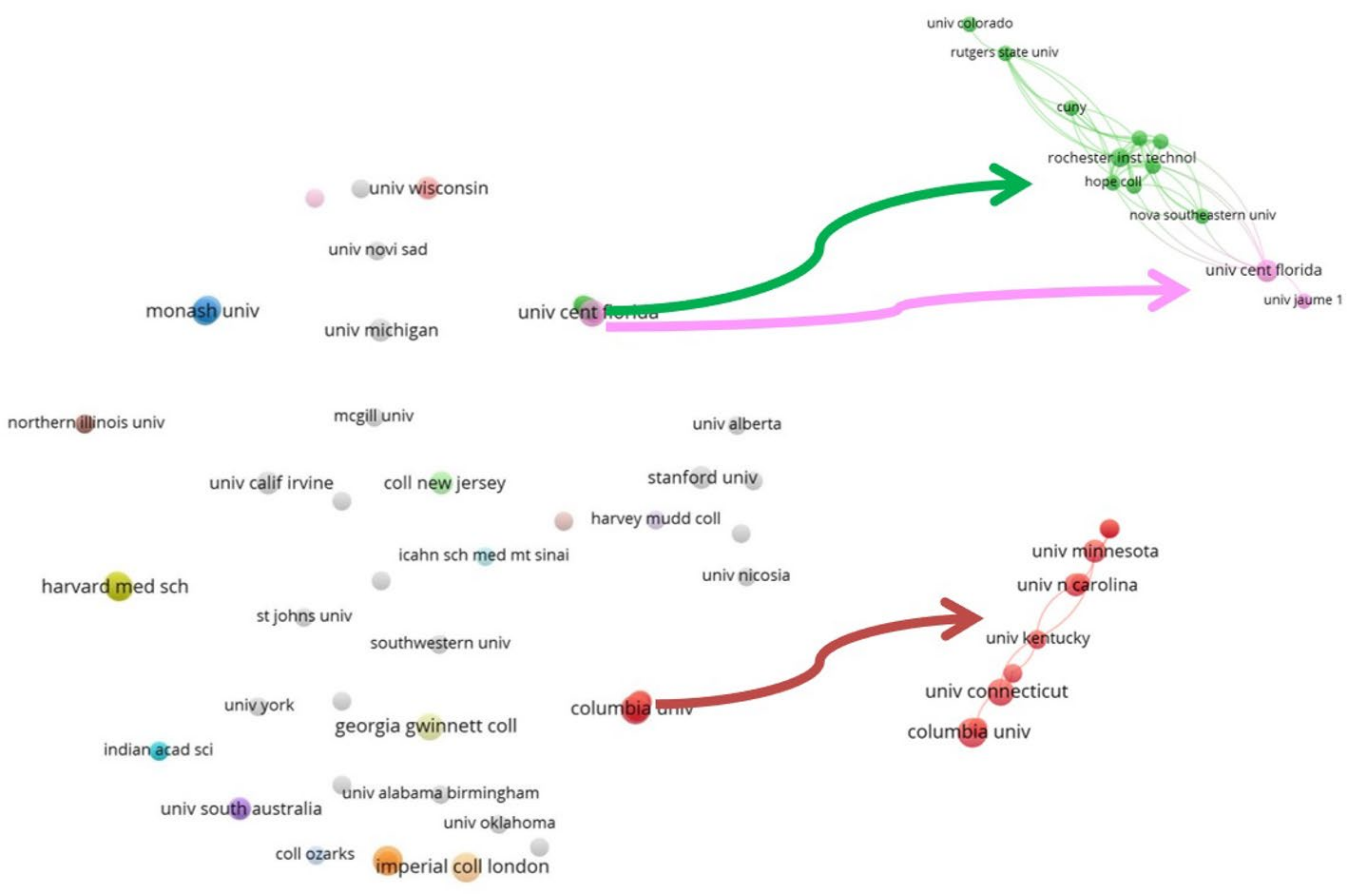
Co-authorship network based on the field ‘organisations’

It is more common to find authors who have published on the subject individually. The grey cluster in Fig. 8 is formed by the researchers A. Salimi and H. ElHawary of McGill University (Montreal); the blue cluster is formed by the researchers B. Chan and G. Lynn, both of whom belong to the College of New Jersey; in the case of the purple cluster, the researchers M. Kemp and H. Alam are affiliated with the University of Michigan; the same is true for the researchers J. Nguyen and K. Keuseman of the orange cluster, who both belong to Mount Mercy University (Iowa); finally, we find the same trend in the green cluster made up of the researchers S. Cahill, P. E. Bergstrom, A. F. Worrall and M. I. Stewart of the University of Oxford. It can be seen that everything is confined to the scope of authors, not only from the same country or the same city, but also from the same institution. This trend is only altered by the yellow and dark blue clusters in Fig. 8. The first is the yellow cluster formed by University of South Australia researchers A. Hofmeyer and K. Kennedy and University of Aberdeen researcher R. Taylor; and also the dark blue cluster with the researchers S. Ramani, J. Cleland, J. Mckimm and R. Fuller, who belong to Harvard Medical School, the University of Aberdeen, Swansea University and the University of Liverpool, respectively. The same trend holds true when it comes to establishing relationships between the main institutions that have produced research on this topic during this period. There is very little collaboration between institutions, except in isolated cases as can be seen in Fig. 9 when looking at the green, pink and red clusters.

**Fig. 10:**
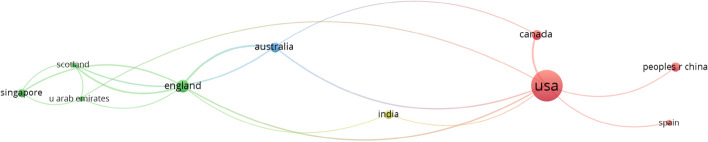
Co-authorship network based on the field ‘countries’

**Fig. 11:**
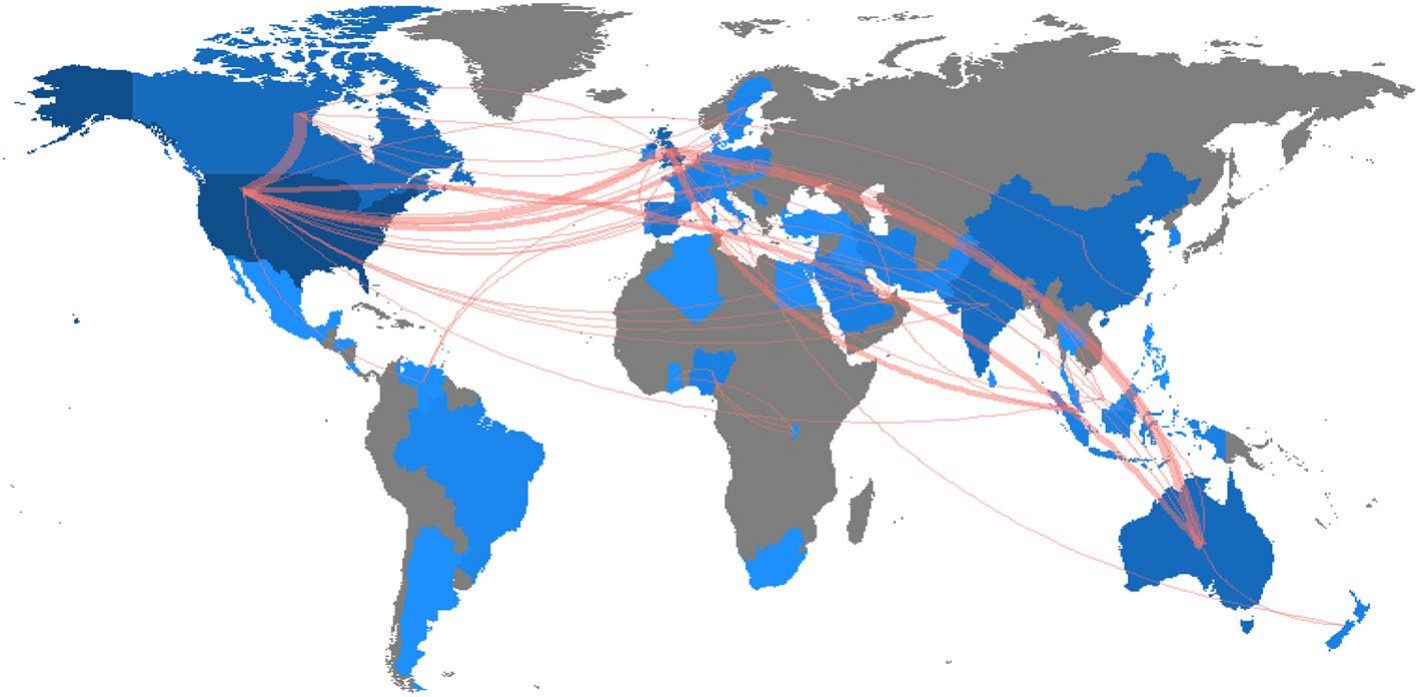
Countries collaboration map

Finally, in terms of map parameters, the minimum number of edges is 1 and in terms of graph parameters the minimum edge size is 5. There is a slightly more noteworthy collaboration between countries, given that to be taken into account a country had to have produced at least five scientific papers. The USA is by far the country with the largest production and the most collaborations (Figs. 10 and 11). It is followed at a considerable distance by countries such as Singapore, England, Australia, India, Canada and China. On the bottom rung are the countries of Scotland, the United Arab Emirates and Spain.

## Discussion and conclusions

The research objectives proposed have been fully achieved. The three types of knowledge structures proposed (conceptual, intellectual and social) have been analysed, with delimitation of the main themes and trends in scientific production on COVID-19 in educational research during 2020 based on different co-occurrence analyses of keywords and factorial solutions.

**Fig. 12:**
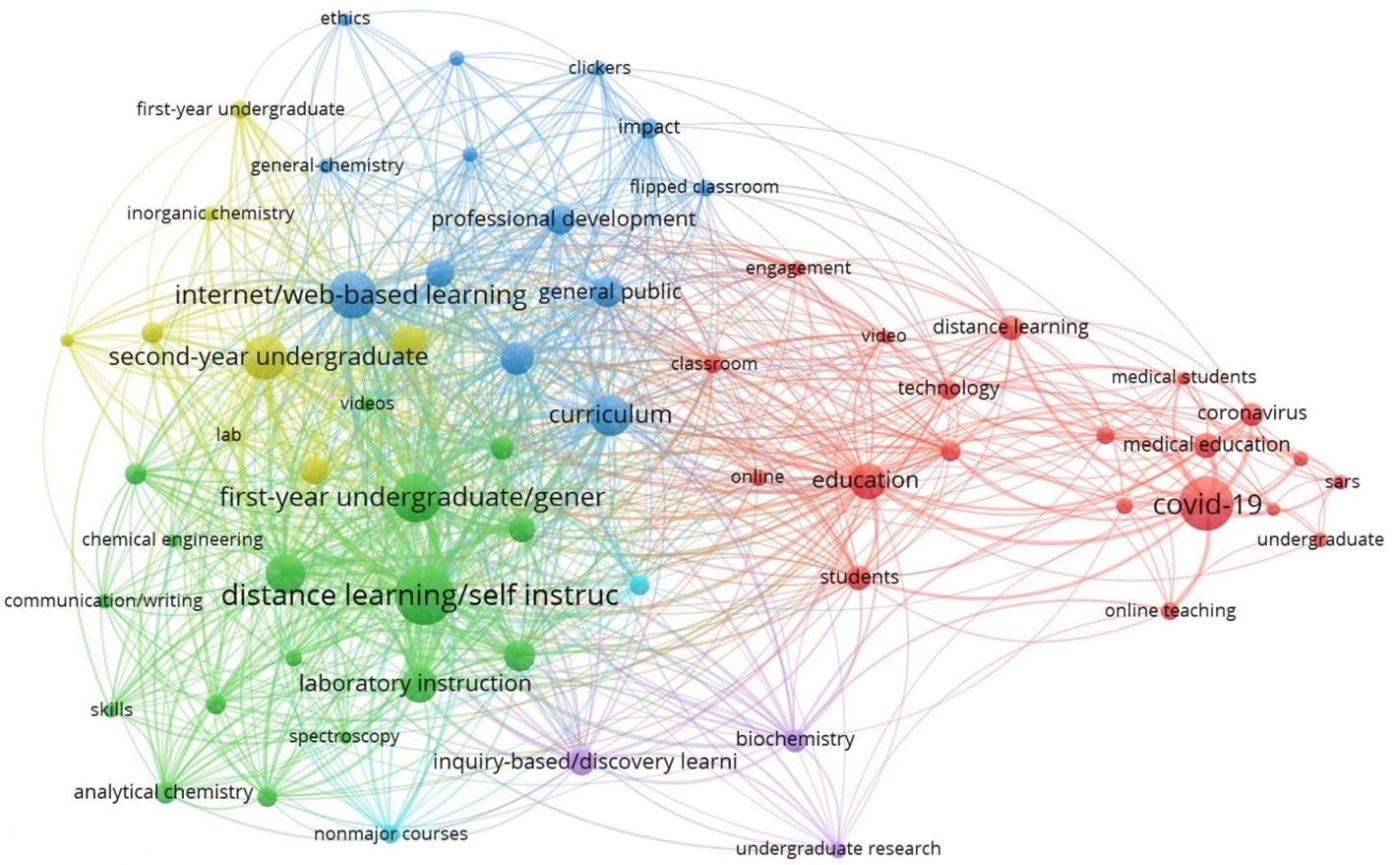
Network visualisation based on all keywords of documents

**Fig. 13:**
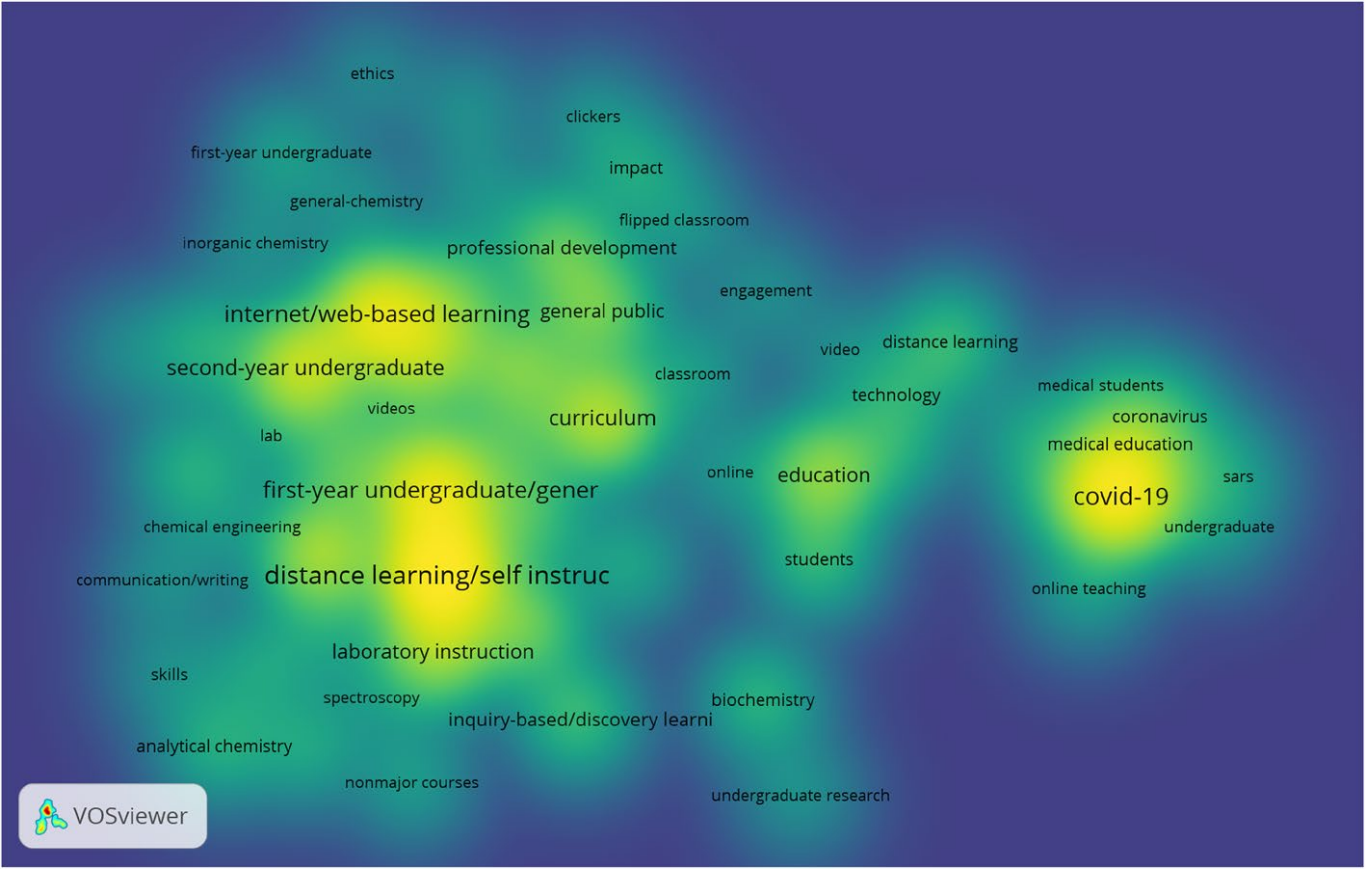
Network visualisation based on all keywords of documents

Following this study of conceptual structure based on a co-occurrence analysis of all keywords, a clear thematic trend may be inferred embracing all matters related with distance education, with a major emphasis on technology and virtual environments for the establishment of new teaching–learning methodologies and procedures brought about by the COVID-19 pandemic situation during 2020. Some of the themes and/or keywords with the highest occurrence values and the highest total link strength (Fig. 12, Table 2), as well as the highest density and relevance in the network (Fig. 13), are *covid-19*; *education*; *internet/web-based learning*; *curriculum*; *second-year undergraduate*; *first-year undergraduate/general*; *distance learning/self instruction* and *laboratory instruction*. Subsequently, a factorial approach was carried out with the idea of reducing and representing the keywords in dimensions or groups of keywords according to their proximity or distance. For this purpose, the keywords plus of the articles were considered. It has been possible to establish two dimensions, one of which is made up of 18 keywords plus (blue cluster) and the other made up of 7 keywords plus (red cluster). The blue cluster, according to its keywords plus, has been called *chemistry science*, assuming particular importance and consistency when it was subsequently verified using the thematic map (Fig. 3) that its terms identify some of the bubbles highlighted according to measurements of centrality and density and the sum of frequencies in terms of their co-occurrence. Meanwhile, the red cluster focuses more on methodologies, evaluation and educational components closer to the reality of educational practice. We finished by analysing the thematic map corresponding to Fig. 3, which established a total of 6 bubbles labelled with the keywords plus with higher co-occurrence value and located on the map according to centrality and density measurements. This allowed establishment of relationships of external connectivity with other neighbouring nodes and internal connectivity between keywords plus that make up the cluster or bubble. According to their position in the different quadrants, the themes were identified as *emerging themes, highly developed and isolated* and *basic and transversal*. Considering the thematic map, including both its bubbles and the terms that make them up, and with a view to establishing relationships with the rest of the analysis and findings, aspects related with the new models of online teaching and structural changes in the curriculum continue to gain strength, with the presence of terms such as *education, curriculum, online, technology, video* and *flipped classroom*. The main stakeholders, apart from teachers, are the students, with notable terms such as *students, skills, student performance, engagement* and *performance*, with a certain focus on performance. Finally, terms perhaps more distanced from the educational sphere such as *chemistry, general-chemistry* and *lab* are indicative of a direct relationship with medical research.

With reference to the intellectual structure, the most outstanding authors in Fig. 4 in terms of co-citation values are found in the blue, green and red clusters. In all cases the studies focus on chemistry as a discipline, addressing aspects such as new strategies and methodologies for chemistry teaching–learning and the evaluation of chemistry as an educational discipline in times of the COVID-19 pandemic. This finding is closely related to the co-cited sources (Fig. 6). The Journal of Chemical Education is the core publication, which forms a satellite network together with other journals of a similar editorial line focusing on study and research of the relationship between chemistry and education, again, in times of the pandemic. This section culminated by highlighting the significant lack of collaboration between institutions at an international level, although not at an intranational level. Several institutions and universities could be organised into three distinct contextual blocks: Australia-New Zealand, Australia and North America (USA).

The social structure consisted of general data revealing limited relationships and collaboration between authors, institutions and countries. When it comes to research produced in the wake of the global pandemic caused by COVID-19, it is striking how few relationships and collaborations there are. One possible explanation for this may lie in the lack of international (mainly political) consensus when seeking to tackle the virus with effective measures, even with the advent of the first vaccines. Each country has ended up adopting a series of general measures, although cities have been able to establish more specific measures at a local level. This may in some way have influenced the establishment of relationships with other authors, institutions or countries. Collaborations between authors have been very limited and, in any case, almost entirely restricted to the local sphere. The findings show that most of the collaborations were between university colleagues, with a reduction of internationalisation to only two clusters, the yellow and dark blue clusters in Fig. 8. In the case of collaborations between institutions, these were between universities in the same country, in this case the USA, as can be seen in the green, pink and red clusters in Fig. 9. In terms of relations established between countries, it is clear that the USA monopolises both research production and collaborations with other countries, especially those with which it shares the same language, such as Australia, Canada or England. In any event, cross-country collaborations are below what might have been expected at the outset.

## Limitations

Finally, no major methodological or analytical limitations were encountered in carrying out this study. However, due to the intention of this research to offer an overview of the state of scientific production around COVID-19 within the educational scientific community, it has not been possible to make a deeper incursion into any of the different knowledge structures analysed, as the research would have been too extensive and dense in its content. This opens the door to future research in which a new and larger sample of data can be collected to cover a longer time spectrum and delve deeper into more specific aspects of some of the existing scientific knowledge structures.

## References

[CR1] Allen J, Rowan L, Singh P (2020). Teaching and teacher education in the time of COVID-19. Asia-Pac. J. of Teach. Educ..

[CR2] Aria M, Cuccurullo C (2017). Bibliometrix: an R-tool for comprehensive science mapping analysis. J. Informetrics..

[CR3] Aria M, Cuccurullo C, D’Aniello L, Misuraca M, Spano M (2022). Thematic Analysis as a New Culturomic Tool: The Social Media Coverage on COVID-19 Pandemic in Italy. Sustainability.

[CR4] Beaunoyer E, Dupéré S, Guitton MJ (2020). COVID-19 and digital inequalities: reciprocal impacts and mitigation strategies. Computers Hum. Behav..

[CR5] Börner, K., Chen, C., & Boyack, K. Visualizing knowledge domains. Annu. Rev. Inf. Sci. Tech. (2005). 10.1002/aris.1440370106

[CR6] Burton DR, Walker LM (2020). Rational vaccine design in the time of COVID-19. Cell Host Microbe.

[CR7] Caddy S (2020). Developing a vaccine for covid-19. Br. Med. J. (BMJ)..

[CR8] Chahrour M, Assi S, Bejjani M, Nasrallah A, Salhab H, Fares M, Khachfe HH (2020). A bibliometric analysis of COVID-19 research activity: a call for increased output. Cureus..

[CR9] Cobo MJ, López-Herrera AG, Herrera-Viedma E, Herrera F (2001). An approach for detecting, quantifying, and visualizing the evolution of a research field: a practical application to the Fuzzy Sets Theory field. J. of Informetr..

[CR10] Corell-Almuzara A, López-Belmonte J, Marín-Marín JA, Moreno-Guerrero AJ (2021). COVID-19 in the Field of Education: State of the Art. Sustainability..

[CR11] Cox M, Cox T, Chen C, Härdle W, Unwin A (2008). Multidimensional Scaling. Handbook of Data Visualization.

[CR12] Cuccurullo, C., Aria, M., & Sarto, F. Foundations and trends in performance management. A twenty-five years bibliometric in business and public administration domains. Scientr. (2016). 10.1007/s11192-016-1948-8

[CR13] Dhawan S (2020). Online learning: a panacea in the time of COVID-19 crisis. J. Educ. Tech. Syst..

[CR14] Drane CF, Vernon L, O’Shea S (2021). Vulnerable learners in the age of COVID-19: a scoping review. Aust. Educ. Res..

[CR35] De la Rosa Troyano, F. F., Martínez Gasca, R., González Abril, L., & Velasco Morente, F.: Análisis de redes sociales mediante diagramas estratégicos y diagramas estructurales. Redes. Rev. Hisp. para el Anál. de Redes Soc. (2005) 10.5565/rev/redes.65

[CR16] Falagas ME, Pitsouni EI, Malietzis GA, Pappas G (2008). Comparison of Pub Med, Scopus, Web of Science, and Google Scholar: strengths and weaknesses. FASEB J..

[CR17] Franceschini F, Maisano D (2012). Quality & Quantity journal: a bibliometric snapshot. Qual. Quant..

[CR18] Garfield E (2004). Historiographic mapping of knowledge domains literature. J. Inf. Sci..

[CR19] Giannakos, M. N., Krogstie, J., & Aalberg, T: Toward a learning ecosystem to support flipped classroom: a conceptual framework and early results. In: Li Chang, Y., Kravcik, M., Popescu, E., Huan, R. & Chen, K. N. S. (eds.), *State-of-the Art* , pp. 105–114. Singapore: Springer (2016).

[CR20] Harrison EA, Wu JW (2020). Vaccine confidence in the time of COVID-19. Eur. J. of Epidemiol..

[CR21] Husson, F. & Josee, J.: Multiple Correspondence Analysis. In: Blasius, J. & Greenacre, M. (eds.) The Visualization and Verbalization of Data. Multiple Correspondence Analysis. pp. 164–181. Boca Raton: CRC Press (2014).

[CR22] Husson, F., Le, S.,& Pagès, J: *Exploratory Multivariate Analysis by Example Using R*. 2nd ed. Boca Raton, Florida: Chapman; Hall/CRC (2017)

[CR23] Jaeger MM, Blaabaek EH (2020). Inequality in learning opportunities during Covid-19: evidence from library takeout. Res. Soc. Stratif. and Mobil..

[CR24] Khaldi H, Prado-Gascó V (2021). Bibliometric maps and co-word analysis of the literature on international cooperation on migration. Qual Quant..

[CR25] Kulkarni AV, Aziz B, Shams I, Busse JW (2009). Comparisons of citation in Web of Science, Scopus and GoogleScholar for articles published in general medical journals. JAMA.

[CR26] López-Belmonte, J., Moreno-Guerrero, A.-J., Pozo-Sánchez, S., & Marín-Marín, J.A.: Co-word analysis and academic performance from the Australasian Journal of Educational Technology in Web of Science. Austr. J. Educational Tech. (2021). 10.14742/ajet.6940

[CR27] Lorenzo G, Gilabert A, Lledó A, Lorenzo-Lledó A (2022). Analysis of Trends in the Application of Augmented Reality in Students with ASD: Intellectual, Social and Conceptual Structure of Scientific Production Through WOS and Scopus. Tech Know Learn..

[CR28] Lotka AJ (1926). The frequency distribution of scientific productivity. J. Wash. Acad. of Sci..

[CR29] Marín-Marín JA, Moreno-Guerrero AJ, Dúo-Terrón P, López-Belmonte J (2021). STEAM in education: a bibliometric analysis of performance and co-words in Web of Science. Int. J. STEM Educ..

[CR30] McCain, K. W.: Mapping economics through the journal literature-an experiment in journal co-citation analysis. J. Am. Soc. Inf. Sci.,** 42**(4), 290–296 (1991)

[CR31] Miguel, S., Moya-Anegón, F., & Herrero-Solana, V.: El análisis de co-citas como método de investigación en Bibliotecología y Ciencia de la Información. Inv. Bibliotecol. (2007). 10.22201/iibi.0187358xp.2007.43.4129

[CR32] Morris, S., & Van Der Martens, B.: Mapping research specialties. Annual Rev. Inf. Sci. Tech., **37**. (2008). 10.1002/aris.2008.1440420113

[CR33] Nguyen MH, Pham TH, Ho MT (2021). On the social and conceptual structure of the 50-year research landscape in entrepreneurial finance. SN Bus Econ..

[CR34] Peters H, Van Raan A (1991). Structuring scientific activities by co-author analysis: An expercise on a university faculty level. Scientometrics.

[CR36] Small H (1973). Co-citation in the scientific literature: a new measure of the relationship between two documents. J. Am. Soc. Inf. Sci..

[CR37] Small H (1997). Update on science mapping: Creating large document spaces. Scientometrics.

[CR38] Van Eck NJ, Waltman L (2010). Software survey: VOSviewer, a computer program for bibliometric mapping. Scientometrics.

[CR15] Van Eck, N. J., & Waltman, L: *VOSviewer Manual: Version 1.6.5*. online: https://www.vosviewer.com/download/f-33s2.pdf (2020). Accessed 26 May 2020

[CR39] Waltz Comarú, M., Matos Lopes, R., Maciel Braga, L.A. Batista Mota, F., & Galvão, C.: A bibliometric and descriptive analysis of inclusive education in science education, Stud. Sci. Educ. (2021). 10.1080/03057267.2021.1897930

